# A differential equation-driven update strategy for density-based topology optimization: implementation with MATLAB codes

**DOI:** 10.1007/s00366-025-02237-6

**Published:** 2026-02-02

**Authors:** Yang Liu, Wei Tan

**Affiliations:** School of Engineering and Materials Science, Queen Mary University of London, London, E1 4NS UK

**Keywords:** Topology optimization, Density method, Differential equation, Update scheme, MATLAB code

## Abstract

Differential equation-driven evolution strategies are often associated with boundary-driven topology optimization methods, such as the level set method. However, differential equations can also be utilized effectively in density-based approaches. This paper presents a design update scheme formulated using differential equations to evolve elemental densities in topology optimization. The proposed scheme transforms the differential equation into an absolute increment format, closely related to the optimality criteria (OC) method, which is traditionally implemented in a relative increment format in density-based methods. The relative increment format of the OC method typically ensures an efficient and stable optimization process, whereas the absolute increment format tends to enable a more active and responsive optimization process, potentially leading to optimized results with improved performance. Furthermore, the absolute increment format can be converted into a relative one if needed. This study explores compliance minimization problems for both isotropic composite and single-material cases. Detailed MATLAB implementations for these cases are presented and thoroughly explained. Numerical examples demonstrate that the differential equation-driven update scheme effectively addresses density distribution optimization problems, offering an alternative to classical density methods.

## Introduction

In recent decades, structural topology optimization has developed rapidly, and researchers have gradually realized that various methods of structural topology optimization share more commonalities than differences [3, 28]. Researchers strive to deepen the theoretical foundation of different methods by learning from and referencing each other. The mutual borrowing and integration among various methods have also facilitated their individual advancements [34, 36].

Based on different ways of structural description, existing structural topology optimization methods can generally be categorized into three branches: methods based on density description [4, 7, 8, 11, 14, 25, 27, 32, 33, 39, 45], methods based on boundary evolution description [1, 2, 18, 20, 21, 23, 26, 31, 35, 37, 41], and methods based on geometric component description [5, 10, 13, 22, 43, 46]. Among these methods, the density-based method and the level set method have long been regarded as two distinct and widely used approaches. In the density method, material distribution is described using continuous element densities. In contrast, the level set method describes material distribution by slicing a high-dimensional surface and mapping it to a lower-dimensional space. In terms of design updates, the density-based method typically uses gradient-based algorithms, such as the Optimality Criteria (OC) method [4, 7, 11, 27], to update the element densities that describe the material distribution. On the other hand, the level set method defines boundary evolution velocities based on shape derivatives. It employs the Hamilton-Jacobi equation to drive the evolution of the cutting boundary of the high-dimensional surface, thereby updating the material distribution. The evolution approach based on differential equations is not unique to the level set method and its variants. For instance, the phase field method and related variants also use differential equations to update material distribution. In fact, the density-based method can also adopt a differential equation framework to implement design updates.

This paper proposes updating the material distribution in density-based methods using a differential equation-driven evolution approach. In density-based methods [4, 11, 14, 25, 27, 32, 33, 39], the definition of material distribution differs. A density value of 0 for an element signifies the absence of material, while a density value of 1 represents solid material. Due to the allowance for continuous variation of element densities in these methods, materials with intermediate density values correspond to composite materials with distinct properties. Similar to the traditional level set method [1, 2, 21, 26, 35], a high-dimensional hypersurface is introduced to represent the material density distribution. By establishing an evolution equation for this hypersurface, a velocity field based on element sensitivity is defined. This velocity field drives the hypersurface’s evolution, thereby optimizing the objective function under given constraints. Unlike the level set method, the hypersurface used in this study is of a higher dimensionality than that in the level set method. Specifically, the level set method employs a three-dimensional surface to describe two-dimensional (2D) structures (or a four-dimensional surface for three-dimensional (3D) structures). In contrast, this study utilizes a four-dimensional function to represent 2D structures (and a five-dimensional function for 3D structures). Furthermore, in the evolution control equation, the evolution velocity of the hypersurface is decomposed into tangential and orthogonal components. By discarding the tangential component and evolving the hypersurface solely based on the orthogonal component, this approach enables the realization of arbitrary topological configurations in the design space. Based on the convergence conditions established through the optimality criteria, the differential equation evolving with the orthogonal velocity component can be discretized and reformulated into an absolute increment format of the optimality criteria (OC) method. In traditional density-based methods, the OC-based iterative format typically adopts a relative increment approach. The relative increment format facilitates an efficient and stable optimization process. In contrast, the absolute increment format responds more directly and sensitively to optimization updates, often helping to escape local optima and evolve toward improved material distributions. However, this advantage often comes at the cost of numerical instability. Setting aside these aspects, the absolute increment format based on the optimal criteria method can be transformed into a relative increment format through reformulation. Therefore, the absolute increment format presented in this paper serves as an effective complement to the OC method.

For educational purposes, this paper provides MATLAB code for a simple problem. Sharing and explaining educational codes for theoretical problems can help researchers gain a deeper understanding of theoretical approaches. Currently, many excellent educational codes for various topology optimization methods, such as methods based on density description [4, 11, 12, 15–17, 27, 33, 38], methods based on boundary evolution description [9, 24, 37, 40], and methods based on geometric component description [10, 30, 42, 44], have been made publicly available, and review articles on these codes have been published in the literature [34, 36]. This has significantly facilitated the exchange and dissemination of various topology optimization methods. In this paper, MATLAB codes are developed to address the distinctive material distribution patterns observed in density-based methods. The codes implement density updates based on differential equations, accommodating two material types: composite materials with intermediate density values and single materials without intermediate values. For the composite material optimization code, its structure aligns with the well-known 99-line [27] and 88-line [4] MATLAB codes for compliance minimization, with the main distinction being in the update iteration format. In contrast, the single-material optimization code features several unique aspects, including the use of a different sensitivity filtering approach, a distinctive hypersurface regularization strategy, and alternative initial structure and volume constraint settings. A series of numerical examples and extensions demonstrate the effectiveness of the proposed update scheme and the developed codes.

The remainder of this paper is organized as follows: Sect. 2 an overview of the whole optimization problem and solving strategy; Sect. 3 introduces the MATLAB implementation of the method with detailed explanation of the code; Sect. 4 presents the numerical examples and how the optimized results are obtained using the developed MATLAB code; followed by discussions on the numerical instability issues using the differential equation in Sect. 5; and finally, Section 6 provides the conclusion of the paper.

## Overview of the optimization problem

### Equilibrium condition

For a 2D linear elasticity problem defined over a bounded open domain $\varOmega \subset \mathbb {R}^{\text {2}}$, the objective is to determine the displacement field $\textbf{u}$ within the material domain $\varOmega$. The displacement field must satisfy the governing equilibrium equations as well as the prescribed boundary conditions on the boundary $\partial \varOmega$. The equilibrium equations are formulated as:

1 $$\begin{aligned} {\left\{ \begin{array}{ll} \nabla \mathbf {\sigma }=\textbf{G} & \text {in }\varOmega \\ \textbf{u}=0 & \text {on }\varGamma _{D}\\ \mathbf {\sigma }\cdot \textbf{n}=\textbf{F} & \text {on }\varGamma _{N} \end{array}\right. } \end{aligned}$$

where $\mathbf {\sigma }$ is the stress tensor, $\textbf{G}$ denotes the body force, $\textbf{n}$ is the outward unit normal to the boundary, and $\textbf{F}$ represents the applied traction. The domain boundary $\partial \varOmega$ is partitioned into two disjoint components: $\partial \varOmega =\varGamma _{\mathcal {D}}\bigcup \varGamma _{\mathcal {N}}$. Specifically, $\varGamma _{\mathcal {D}}$ corresponds to the region where Dirichlet boundary conditions are prescribed, while $\varGamma _{\mathcal {N}}$ denotes the portion of the boundary subject to Neumann boundary conditions.

### Material distribution function

In the density-based methods, the design variables are elemental densities, and the material distribution is represented by the distribution of the density field. This density field can be described by a high-dimensional hypersurface $\phi$, where $\phi$ serves as a underlying surrogate model used to update the density field during the optimization process.

Let $\phi ^{*}\left( \textbf{x}\right)$: $\mathbb {R}^{n+1}\rightarrow \mathbb {R}$ denote a smooth multivariate function defined on an $\left( n+1\right)$-dimensional ambient space, where $\textbf{x}=\left( \textbf{x}^{\mathcal {T}},x^{\mathcal {O}}\right)$. The domain $\mathbb {R}^{n+1}$ is decomposed into two orthogonal subspaces:

- Tangent space $\mathcal {T}$: An *n*-dimensional subspace spanned by local coordinates $\textbf{x}^{\mathcal {T}}=\left( x_{1},x_{2},\cdots ,x_{n}\right)$.
- Orthogonal space $\mathcal {O}$: A 1-dimensional complementary subspace spanned by coordinates $x^{\mathcal {O}}=\left( x_{n+1}\right)$.

A function $\phi \left( \textbf{x}^{\mathcal {T}},x^{\mathcal {O}}\right) =\phi ^{*}\left( \textbf{x}^{\mathcal {T}},x^{\mathcal {O}}=f\left( \textbf{x}^{\mathcal {T}}\right) \right)$:$\mathbb {R}^{n+1}\rightarrow \mathbb {R}$ is termed embedded reference hypersurface, where *f*:$\mathcal {T}\rightarrow \mathbb {\mathcal {O}}$ is a bounded, continuous, and injective embedding functions that parameterize the hypersurface in the orthogonal space $\mathbb {\mathcal {O}}$.

Let $\rho$: $\mathbb {R}^{n+1}\rightarrow \left\{ f_{\rho },0\right\}$ be the material distribution function for a structure, where the projection $f_{\rho }$ is a function of the hypersurface $\phi$. The specific expression of $\rho$ takes the following:

2 $$\begin{aligned} \rho \left( \phi \right) ={\left\{ \begin{array}{ll} f_{\rho }\left( \phi \right) & \phi \left( \textbf{x}^{\mathcal {T}},x^{\mathcal {O}}\right) \ge 0\quad \forall \textbf{x}^{\mathcal {T}}\in \varOmega \cup \partial \varOmega \\ 0 & \phi \left( \textbf{x}^{\mathcal {T}},x^{\mathcal {O}}\right) <0\quad \forall \textbf{x}^{\mathcal {T}}\in D\setminus \varOmega \end{array}\right. } \end{aligned}$$

where $\rho$ is a non-negative, continuous, and differentiable material distribution function; $\varOmega$ denotes the material region within the design domain *D*, and $\partial \varOmega$ represents its material interface. The material distribution $\rho$ depends on the material composition: for single-material systems, $\rho$ is typically constant (implemented via a Heaviside projection of $f_{\rho }$), whereas for isotropic composite materials, $\rho$ becomes spatially variable (characterized by a continuous projection of $f_{\rho }$).

**Fig. 1:**
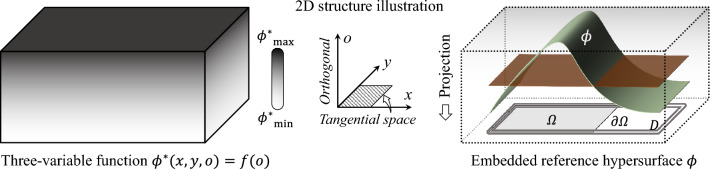
Schematic illustration of material distribution description for 2D structures. $\phi ^{*}$ is a ternary function residing in a four-dimensional space. By referencing the general evolution of a hypersurface $\phi$ and projecting it orthogonally onto the structural plane, $\phi$ can describe changes in structural shape and topology

For example, for 2D structures, let $\textbf{x}^{\mathcal {T}}=\left( x,y\right)$ and $x^{\mathcal {O}}=\left( o\right)$. Let $\phi ^{*}\left( x,y,o\right)$ be a three-variable function, and let $\phi \left( x,y,o\right) =f\left( o\right)$. As illustrated in Fig. 1, the positional relationship between $\phi$ and the structural plane can describe the material distribution of arbitrary 2D structures under orthographic projection. Note that for a 2D structural problem, the hypersurface $\phi$ resides in a four-dimensional ambient space, where $\textbf{x}\subset \mathbb {R}^{\text {3}}$; for a 3D structural problem, $\phi$ evolves into a five-dimensional function, with $\textbf{x}\subset \mathbb {R}^{\text {4}}$.

**Fig. 2:**
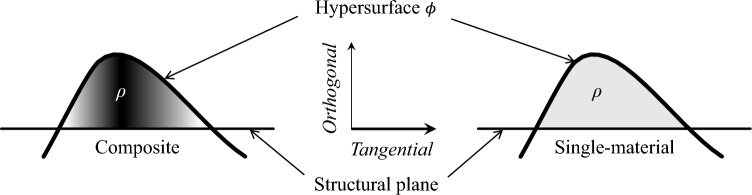
Illustration of material distribution defined through a high-dimensional hypersurface for various forms of material composition. The variable density field $\rho$ corresponds to composite materials, while the constant density field $\rho$ represents single-material configurations

By performing orthogonal projection of the hypersurface $\phi$, the density field $\rho$ can be mapped to the structural plane. The material distribution $\rho$ depends on the material composition. As depicted in Fig. 2, for single-material systems, $\rho$ is typically constant (implemented via a Heaviside projection of $f_{\rho }$), whereas for isotropic composite materials, $\rho$ becomes variable (characterized by a continuous projection of $f_{\rho }$).

### Evolution governing equation

As discussed above, the density field $\rho$ is determined by the hypersurface $\phi$, and its variation is governed by the evolution of $\phi$. By introducing a pseudo-time parameter *t*, the density field $\rho$ become time-dependent, expressed as $\rho \left( \phi \left( \textbf{x}\left( t\right) ,t\right) \right)$. At the convergence of the optimization process, the time derivative of $\rho$, denoted as $\rho _{t}^{\prime }$, becomes zero. This leads to the following stationary equation:

3 $$\begin{aligned} \frac{\partial \rho }{\partial \phi \left( \textbf{x}\left( t\right) ,t\right) }\left( \frac{\partial \phi \left( \textbf{x}\left( t\right) ,t\right) }{\partial t}+\nabla \phi \left( \textbf{x}\left( t\right) ,t\right) \cdot \frac{\partial \textbf{x}\left( t\right) }{\mathrm {\partial }t}\right) =0 \end{aligned}$$

This stationary equation indicates that the material distribution becomes time-independent at convergence. Two key implications arise from this insight:

1. For composite materials, $\partial \rho /\partial \phi \ne 0$, ensuring that the second term in Eq. (3) above equals zero.
2. For single materials, $\partial \rho /\partial \phi =0$. In this case, as the material distribution ceases to evolve at convergence, the material boundary (where $\phi =0$) also becomes time-independent, implying $\phi ^{\prime }\left( \textbf{x}\left( t\right) ,t\right) =0$. Consequently, the second term in the stationary equation Eq. (3) also remains zero.

Based on the above, the evolution equation governing the hypersurface $\phi$ is independent of material composition and can be expressed as:

4 $$\begin{aligned} \frac{\partial \phi }{\partial t}+\nabla _{\textbf{x}^{\mathcal {T}}}\phi \cdot \frac{\partial \textbf{x}^{\mathcal {T}}}{\mathrm {\partial }t}+\nabla _{x^{\mathcal {O}}}\phi \frac{\partial x^{\mathcal {O}}}{\mathrm {\partial }t}=0 \end{aligned}$$

**Fig. 3:**
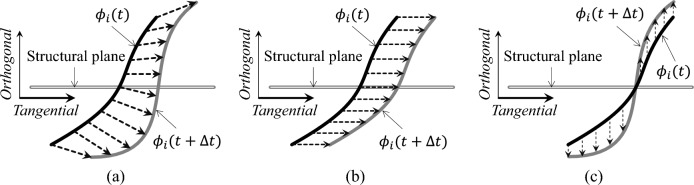
Illustration of material distribution variations driven by hypersurface evolution. **a** Generalized evolution of hypersurface. **b** Evolution of hypersurface along the defined tangential direction. **c** Evolution of hypersurface along the defined orthogonal direction

Equation (4) represents the generalized evolution of $\phi$, enabling any potential change in material distribution [19]. Let $\bar{\textbf{v}}=\left( \bar{\textbf{v}}^{\mathcal {T}},\bar{v}^{\mathcal {\mathcal {O}}}\right)$ denote the velocity field driving the evolution of $\phi$, where $\bar{\textbf{v}}^{\mathcal {T}}=\partial \textbf{x}^{\mathcal {T}}/\mathrm {\partial }t$ is the tangential velocity, and $\bar{v}^{\mathcal {\mathcal {O}}}=\partial x^{\mathcal {O}}/\mathrm {\partial }t$ is the orthogonal velocity. The generalized evolution manner of $\phi$ is illustrated in Fig. 3a. As shown, the generalized evolution can be decomposed into tangential evolution (Fig. 3b) and orthogonal evolution (Fig. 3c). In the conventional level set method, tangential evolution is typically utilized for design updates, with the orthogonal velocity often disregarded. Conversely, in density-based methods, tangential velocities are generally neglected (i.e., $\bar{\textbf{v}}^{\mathcal {T}}=0$), simplifying the governing equation in Eq. (4) to the following:

5 $$\begin{aligned} \frac{\partial \phi }{\partial t}+\nabla _{x^{\mathcal {O}}}\phi \bar{v}^{\mathcal {\mathcal {O}}}=0 \end{aligned}$$

This is the governing differential equation for density-based methods. Equation (5) indicates that the density surface evolves solely through the orthogonal velocity. Through Eq. (5), any topological variation, including nucleation of solid or void regions, can be achieved during the optimization process.

### Optimization formulation

The mathematical formulation of the structural optimization problem involves defining the design variables, objective function, and constraints. In this work, the elemental density $\rho$ is employed as the design variable, while the high-dimensional hypersurface $\phi$ serves as a surrogate model for the structure. The optimization problem can be expressed as follows:

6a $$\begin{aligned} \underset{\mathrm {find:}\rho }{\text {Minimize}} & \mathcal {J}\left( \rho \right) =\sum _{e=1}^{N} \mathop {E\left( \rho \right) \mathrm {\textbf{u}}_{e}^{\textrm{T}}\mathrm {\textbf{k}}_{e}\mathrm {\textbf{u}}_{e}}\end{aligned}$$

6b $$\begin{aligned} \text {Subject\,to}: & \mathrm {\textbf{KU}}=\mathrm {\textbf{F}}\end{aligned}$$

6c $$\begin{aligned} & \mathcal {V}\left( \rho \right) =\mathcal {V}_{0}\mathcal {V}_{f}\end{aligned}$$

6d $$\begin{aligned} & 0\le \rho \le 1 \end{aligned}$$

where $\mathcal {J}$ is the objective function representing structural compliance, *E* is the elemental modulus, $\mathrm {\textbf{u}}_{e}$ is the elemental displacement vector, and $\mathrm {\textbf{k}}_{e}$ is the elemental stiffness matrix. The global stiffness matrix is denoted by $\mathrm {\textbf{K}}$, $\mathrm {\textbf{U}}$ is the global displacement vector, and $\mathrm {\textbf{F}}$ is the force vector. $\mathcal {V}$ represents the volume of the material region, $\mathcal {V}_{0}$ is the total volume of the design domain, and $\mathcal {V}_{f}$ denotes the prescribed material volume fraction.

### Sensitivity

Elemental sensitivity quantifies the influence of each element on the compliance of a structure. It measures the effect of a sufficiently small change $\Delta \rho$ in the material density of an element on the structural performance. For a given objective function, the elemental sensitivity $\textrm{d}_{\rho }\mathcal {J}$ with respect to $\rho$ is formally defined as:

7 $$\begin{aligned} \textrm{d}_{\rho }\mathcal {J}=\underset{\Delta \rho \rightarrow 0}{\text {lim}}\frac{\mathcal {J}\left( \rho +\Delta \rho \right) -\mathcal {J}\left( \rho \right) }{\Delta \rho } \end{aligned}$$

For composite materials, the elemental modulus $E_{C}$ can take the following material interpolation model:

8 $$\begin{aligned} E_{C}\left( \rho \right)= & E_{\min }+\rho \left( E_{0}-E_{\min }\right) \end{aligned}$$

where $E_{\min }$ is a small nonzero value to prevent singular stiffness matrices, and $E_{0}$ represents the stiffness of the fully dense material. Based on this interpolation, the elemental sensitivity $\mathcal {J}_{C}^{\prime }\left( \rho \right)$ for a compliance optimization problem involving composite materials is given by:

9 $$\begin{aligned} \mathcal {J}_{C}^{\prime }\left( \rho \right)= & -\left( E_{0}-E_{\min }\right) \mathrm {\textbf{u}}_{e}^{\textrm{T}}\mathrm {\textbf{k}}_{e}\mathrm {\textbf{u}}_{e} \end{aligned}$$

In such cases, the optimization problem is convex, and the solution is guaranteed to be globally optimal.

However, for single-material models, this interpolation approach can lead to numerical oscillations during optimization. The differential equation update in Eq. (5) for single-material systems only supports material changes along boundaries, requiring significant distinction in the sensitivities of boundary elements. If sensitivities around the boundary are insufficiently distinct, boundary stability issues may arise. To mitigate this, a boundary penalization scheme is commonly used. In density-based methods, a power-law penalization is widely adopted for material interpolation [4, 7, 27], enhancing contrast in sensitivities between material and void regions to stabilize boundary evolution. The penalized stiffness model is expressed as:

10 $$\begin{aligned} E_{S}\left( \rho \right)= & E_{\min }+\left( \rho \right) ^{p}\left( E_{0}-E_{\min }\right) \end{aligned}$$

where $E_{S}$ is the elemental modulus for single-material cases, and *p* is the penalty factor. Penalization ensures that boundary sensitivities are sufficiently distinct, thus stabilizing boundary evolution. For a static compliance problem, the elemental sensitivity $\mathcal {J}_{S}^{\prime }\left( \rho \right)$ under this penalization is:

11 $$\begin{aligned} \mathcal {J}_{S}^{\prime }\left( \rho \right)= & -p\left( \rho \right) ^{p-1}\left( E_{0}-E_{\min }\right) \mathrm {\textbf{u}}_{e}^{\textrm{T}}\mathrm {\textbf{k}}_{e}\mathrm {\textbf{u}}_{e} \end{aligned}$$

Applying penalization transforms the optimization problem into a non-convex one, making the result generally a local optimum.

### Steepest descent method

The hypersurface $\phi$ serves as a surrogate model, analogous to a response surface, facilitating the evolution of material distributions through the differential equation defined in Eq. (5). To ensure that the evolution of $\phi$ in Eq. (5) leads to an optimized material distribution, it is critical to identify a descent direction for the velocity field $\bar{v}^{\mathcal {\mathcal {O}}}$. Using the method of Lagrange multipliers, the constrained optimization problem in Eq. (6a) can be reformulated as an unconstrained problem, expressed through the Lagrangian function $\mathscr {L}$:

12 $$\begin{aligned} \mathfrak {\mathcal {\mathscr {L}}}\left( \rho \right) =\mathcal {J}\left( \rho \right) +\lambda \left( \mathcal {V}\left( \rho \right) -\mathcal {V}_{0}\mathcal {V}_{f}\right) \end{aligned}$$

where $\lambda$ is the Lagrange multiplier associated with the material volume constraint. The time derivative of the Lagrangian, $\mathcal {\mathscr {L}}_{t}^{\prime }$, is given by:

13 $$\begin{aligned} \mathcal {\mathscr {L}}_{t}^{\prime }=\int _{\varOmega }\frac{\mathrm {\partial }\mathcal {\mathscr {L}}}{\mathrm {\partial }\rho }\frac{\mathrm {\partial }\rho }{\mathrm {\partial }t}\textrm{d}\varOmega \end{aligned}$$

To ensure a descent direction for the Lagrangian, the velocity field $\bar{v}^{\mathcal {\mathcal {O}}}$ is defined as:

14 $$\begin{aligned} \bar{v}^{\mathcal {\mathcal {O}}}=\mathcal {\mathscr {L}}_{\rho }^{\prime }=\mathcal {J}_{\rho }^{\prime }+\lambda \mathcal {V}_{\rho }^{\prime } \end{aligned}$$

It can be demonstrated that this formulation ensures a descent direction for the Lagrangian function $\mathcal {\mathscr {L}}$. Rewriting the stationary equation of $\rho \left( \phi \right)$ yields:

15 $$\begin{aligned} \rho _{t}^{\prime }=\frac{\partial \rho }{\partial \phi }\left( \frac{\partial \phi }{\partial t}+\frac{\partial \phi }{\partial x_{\mathcal {O}}}\bar{v}^{\mathcal {\mathcal {O}}}\right) =0 \end{aligned}$$

where $\partial \rho /\partial \phi \ge 0$. Assuming $\partial \phi /\partial x_{\mathcal {O}}\nleq 0$, the term $\partial \phi /\partial x_{\mathcal {O}}$ can be treated as a damping coefficient. Substituting this into the derivative of the Lagrangian function yields the following:

16 $$\begin{aligned} \mathcal {\mathscr {L}}_{t}^{\prime }=\int _{\varOmega }\frac{\mathrm {\partial }\mathcal {\mathscr {L}}}{\mathrm {\partial }\rho }\frac{\partial \rho }{\partial \phi }\frac{\mathrm {\partial }\phi }{\mathrm {\partial }t}\textrm{d}\varOmega =-\int _{\varOmega }\frac{\partial \rho }{\partial \phi }\frac{\partial \phi }{\partial x_{\mathcal {O}}}\left( \frac{\mathrm {\partial }\mathcal {\mathscr {L}}}{\mathrm {\partial }\rho }\right) ^{2}\textrm{d}\varOmega \le 0 \end{aligned}$$

This demonstrates that $\bar{v}^{\mathcal {\mathcal {O}}}=\mathcal {\mathscr {L}}_{\rho }^{\prime }$ provides a descent direction for the optimization problem, ensuring monotonic decrease of the Lagrangian $\mathcal {\mathscr {L}}$. Consequently, the objective function $\mathcal {J}$ evolves in the direction of optimization under Eq. (5).

According to the Karush-Kuhn-Tucker (KKT) conditions, the necessary condition for optimization convergence is given by: $\mathcal {\mathscr {L}}_{\rho }^{\prime }=0$, which implies:

17 $$\begin{aligned} \mathcal {J}_{\rho }^{\prime }+\lambda \mathcal {V}_{\rho }^{\prime }= & 0 \end{aligned}$$

At convergence, this condition indicates that the velocity field $\bar{v}^{\mathcal {\mathcal {O}}}$ of the structure is zero everywhere.

### Design update scheme

As previously discussed, the differential equation in Eq. (5), incorporating the sensitivity-based velocity field $\bar{v}^{\mathcal {\mathcal {O}}}$, guarantees the descent direction for the objective function. By discretizing the pseudo-time *t*, the update scheme of the hypersurface $\phi$ based on Eq. (5) can be expressed as follows:

18 $$\begin{aligned} \Delta \phi =\phi _{i+1}-\phi _{i}=-\Delta t\nabla _{x^{\mathcal {O}}}\phi _{i}\bar{v}^{\mathcal {\mathcal {O}}}=-\Delta t\nabla _{x^{\mathcal {O}}}\phi _{i}\left( \mathcal {J}_{\rho }^{\prime }+\lambda \mathcal {V}_{\rho }^{\prime }\right) \end{aligned}$$

where *i* represents the number of iteration step. The term $\nabla _{x^{\mathcal {O}}}\phi _{i}$, a function of $\phi$ and sensitivity-related variables, can act as a damping coefficient to regulate the optimization process. The Lagrange multiplier $\lambda$ can be calculated using the bisection method [4, 27]. The iterative update scheme in Eq. (18) adopts an absolute increment format of the OC method, differing from the relative increment format commonly used in traditional density-based methods [4, 6, 27]. Generally, the relative increment format of OC ensures a more efficient and stable optimization process, while the absolute increment format of OC tends to enable more active optimization and better captures subtle sensitivity variations. Nevertheless, the absolute increment format can be transformed into a relative increment format. For instance, a relative increment format for Eq. (18) can be given as follows:

19 $$\begin{aligned} \frac{\phi _{i+1}}{\phi _{i}}=1-\frac{\Delta t}{\phi _{i}}\nabla _{x^{\mathcal {O}}}\phi _{i}\bar{v}^{\mathcal {\mathcal {O}}}=B_{e}^{*} \end{aligned}$$

where $B_{e}^{*}$ is a term associated with optimality criteria. When $B_{e}^{*}<1$, $\phi _{i+1}$ reduces; when $B_{e}^{*}>1$, $\phi _{i+1}$ increases; and when $B_{e}^{*}=1$, $\phi _{i+1}$ remain unchanged. This is consistent with the methodology described in [4, 6, 27]. In practical implementation, the damping term $\nabla _{x^{\mathcal {O}}}\phi _{i}$ can be set to 1, effectively disabling damping. In this case, Eq. (18) reduces to the following:

20 $$\begin{aligned} \phi _{i+1}=\phi _{i}-\Delta t\bar{v}^{\mathcal {\mathcal {O}}} \end{aligned}$$

This simplified format is applicable to both composite and single-material scenarios.

The stopping criteria for the optimization process can be defined as either a minimum relative change in the objective function (e.g., compared over the last five iterations) or reaching a user-specified maximum iteration count:

21 $$\begin{aligned} \frac{\left| \mathcal {J}_{i}-\mathcal {J}_{i-5}\right| }{\mathcal {J}_{i}}\le 10^{-4},\quad \text {or}\,i\ge \text {loop}_{\max } \end{aligned}$$

### Filtering

**Fig. 4:**
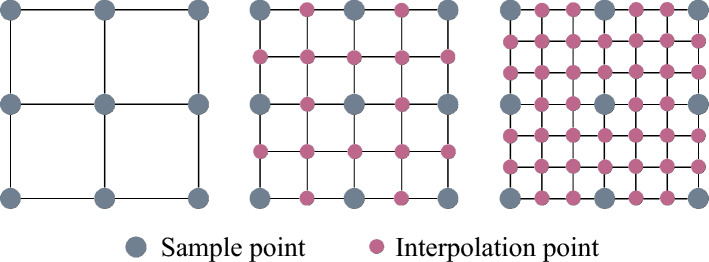
A sampling method for acquiring gradient information. The sampling grid is independent of the FEA mesh; when the analysis mesh changes, the sampling grid can remain unchanged, which helps mitigate mesh dependency in design

In the proposed method, the evolution of the hypersurface $\phi$ relies on a gradient-based velocity field. When constructing this velocity field, the method employs a sampling approach to obtain it. By sampling the computed gradient information, the driving force for hypersurface evolution is constructed. During the sampling process, establishing a sampling grid independent of the FEA mesh can effectively mitigate the mesh dependency issue in design. As illustrated in the Fig. 4, after establishing an interpolation relationship between the sampling grid and the FEA mesh, the sampling grid for gradient information remains unchanged regardless of how the FEA mesh is modified.

In density-based topology optimization methods, filtering techniques are commonly employed to mitigate issues such as checkerboarding and numerical instability. For composite materials, the hypersurface $\phi$ is typically mapped at the element centers, allowing for a continuous variation of material densities. As a result, the need for additional filtering is effectively eliminated. In contrast, for single-material cases, material distribution is defined by the intersection of the hypersurface $\phi$ with the structural plane ($\phi =0$). This approach can lead to checkerboarding artifacts and zigzag boundaries in the final structure. To ensure smoothness and continuity of the structural boundary, the hypersurface $\phi$ must be smooth and continuous. This can be achieved by mapping the hypersurface to the element nodes and constructing a nodal velocity field $\bar{v}_{N}$ through filtering the sensitivities of elements sharing the node. The nodal velocity field is defined using an averaging scheme as follows:

22 $$\begin{aligned} \bar{v}_{N} = \frac{1}{n_{e}} \sum _{j=1}^{n_{e}} \mathcal {J}_{\rho j}^{\prime } \end{aligned}$$

where $n_{e}$ represents the total number of elements connected to a common node, and $\mathcal {J}_{\rho j}^{\prime }$ denotes the sensitivity of the objective function with respect to the density of element *j*. Compared to the widely used convolution-based filtering schemes in traditional density methods, the filtering scheme described in Eq. (22) corresponds to the minimum possible filtering radius. While filtering inevitably constrains the design space to some extent, minimizing the filtering radius helps maintain a broader and more flexible design space, enabling finer control over the optimization process.

### Volume relaxation

The optimization results in topology optimization are often influenced by the choice of the initial design, particularly for non-convex problems. In single-material cases, the initial design must be carefully selected to mitigate the impact of initial conditions on the final solution. A commonly adopted approach is to use a fully materialized initial design, as it ensures the completeness of the solution within the design space. By starting with a full-material design and progressively relaxing the material volume to meet the prescribed volume constraint, the optimization process is decelerated, reducing the risk of premature convergence to suboptimal solutions. The volume relaxation process can be implemented using a linear relaxation scheme expressed as:

23 $$\begin{aligned} G_{i}=\mathcal {V}_{f}+\left( 1-\mathcal {V}_{f}\right) \max \left( 0,1-\frac{i}{N_{r}}\right) \end{aligned}$$

where $G_{i}$ is the material volume in the *i*th step, and $N_{r}$ is the given relaxation step number. As described by Eq. (23), the material volume constraint is gradually reduced in the first $N_{r}$ iterations before reaching the permitted volume fraction. This approach helps to smooth the optimization process, ensuring a more robust convergence by effectively navigating the design space without overly constraining the material layout in the early stages.

For the calculation of element density, a statistic-based point-sampling method can be simply adopted. Specifically, several points are uniformly distributed within the element, the number of points where the $\phi$ value exceeds 0 is counted, and the ratio of this count to the total number of points is computed as the element density value. For elements not intersected by structural boundaries, the density values calculated by this method are exact. For elements intersected by structural boundaries, however, the density values are approximate, depending on the number of sampling points: a greater number of points improves accuracy but simultaneously reduces computational efficiency.

### Regularization

The hypersurface serves as a surrogate model for describing and evolving material distribution during the optimization process. To ensure stable and efficient convergence, it is crucial to maintain an appropriate morphology for the hypersurface, particularly in single-material cases. In composite materials, the elemental density is directly related to the magnitude of the hypersurface value $\phi$. Since the elemental density satisfies the constraint $0\le \rho \le 1$, the values of $\phi$ are also confined within the same bounds. However, in the case of single-material problems, the elemental density depends solely on the sign of $\phi$, meaning that $\phi$ is unbounded in terms of magnitude. As a consequence, there are lower and upper bounds applied to the value of $\phi$. This introduces the potential for instability, as excessive values of $\phi$ can lead to an ill-posed material distribution, where the solid-to-void or void-to-solid transformation may not occur efficiently. To mitigate this issue, a compressing regularization technique is applied to the hypersurface for the single-material case, which is expressed as:

24 $$\begin{aligned} \phi ^{R}=\alpha \phi ,\;\;0<\alpha \le 1 \end{aligned}$$

where $\alpha$ is a compression regularization factor. This approach effectively scales the hypersurface closer to the structural plane, facilitating smoother transitions between solid and void regions. The regularization acts as a linear scaling that modifies the absolute magnitudes of the hypersurface values without affecting the relative proportions of the material distribution. By applying this regularization at appropriate intervals, the hypersurface remains well-behaved throughout the optimization process, enhancing the topology evolution and ensuring the stability of the design. For further details on this regularization strategy, refer to [20].

## MATLAB implementation

### Composite material

This section explains a 44-line MATLAB code designed for compliance minimization of 2D structures with composite materials (refer to Appendix A). The code is executed from the MATLAB prompt using the following command:
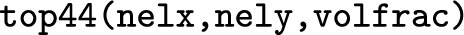


where nelx and nely represent the number of elements in the horizontal and vertical directions, respectively, and volfrac denotes the prescribed volume fraction.

The structure of the MATLAB code can be divided into three main parts:

1. Finite Element Analysis Preparation: This section initializes material properties (line 4) and prepares for the finite element analysis (lines 5–14). Key preprocessing steps for FEA, including element stiffness matrix assembly and boundary connectivity, follow the approach described in [4].
2. Boundary Conditions: The displacement constraints (line 16) and external load conditions (line 18) are specified in this part. These definitions ensure appropriate structural behavior under the given conditions.
3. Optimization Solving: The main optimization process comprises five components:

- Design Initialization: Initializing the material distribution (line 21).
- Structural Response Analysis: Conducting FEA (line 26), computing the objective function (line 28), and evaluating sensitivities (line 29).
- Result Display: Visualization of the current optimization iteration (lines 31–32).
- Design Update: Iterative refinement of the design based on sensitivity analysis (lines 34–41).
- Convergence Check: Ensuring the optimization terminates upon satisfying predefined criteria (line 43).

The overall structure of the code largely follows the framework proposed by Andreassen et al. (2011) [4], with the primary difference being the implementation of the design update scheme. The specific methodology for design update is presented in detail below.
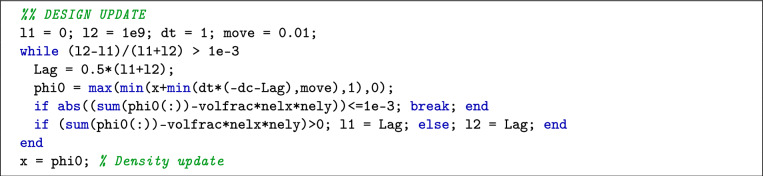


Within the design update process, parameters l1 and l2 are utilized in the bisection method to determine the Lagrange multiplier $\lambda$, which enforces the material volume constraint. The parameter dt represents the prescribed pseudo-time step, and move defines the upper limit for changes in the hypersurface value. Line 37 of the MATLAB code implements the update scheme described in Eq. (20). The choice of dt is critical, as the update scheme represents an absolute increment format of the OC method. If the mean absolute increment of the sensitivity field is relatively small, dt should be set larger to accelerate the optimization evolution. Conversely, if the sensitivity field increment is relatively large, a smaller dt value is advisable to avoid oscillations, given the active nature of the optimization process. In practice, an effective strategy is to start with a relatively large initial value for dt and then gradually reduce it in a continuous manner to achieve a balance between efficiency and stability throughout the optimization process. The parameter move in this study is set smaller than in previous works [4, 27]. This conservative setting serves as an additional measure to suppress potentially overactive updates in the hypersurface values. This combined approach ensures a smoother convergence and enhances the robustness of the optimization process.

### Single-material

For single-material compliance minimization of 2D structures, a 55-line MATLAB code is provided (see Appendix B). The code is executed in the same manner as the composite material case using the following command:
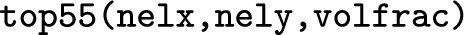


The structure of this 55-line code closely aligns with the 44-line code for composite materials, with key differences found in the OptimizationSolving section. Algorithm-specific parameters are defined as follows:
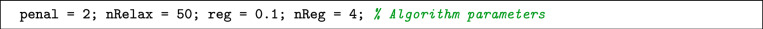


Here, penal represents the penalty factor *p*, nRelax is the relaxation step number $N_{r}$, reg corresponds to the compression regularization factor $\alpha$, and nReg denotes the regularization frequency. In the Structural Response Analysis section, sensitivity filtering is implemented with the following line:
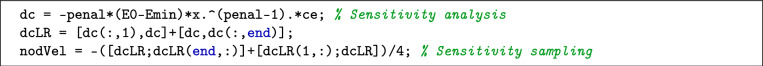


Here, dc is the derivative of the objective function with respect to the element density. dcLR iserves as an intermediate variable for mapping elemental sensitivities to the nodal velocity, and nodVel is the nodal velocity filtered by averaging sensitivities of elements sharing a common node. In the Design Update section, the detailed implementation code is given as follows:
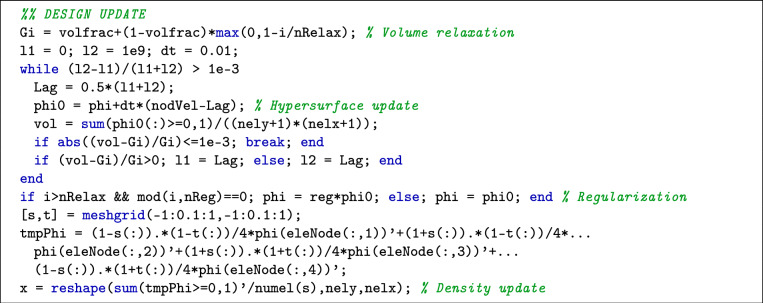


The following components of the Design Update section are noteworthy:

- VolumeRelaxation: Line 38 implements the volume relaxation described in Eq. (23).
- Update Scheme: Line 42 corresponds to the update scheme in Eq. (20). As hypersurface values are unbounded in this implementation, the material distribution depends solely on the hypersurface sign. Consequently, the optimization process demonstrates reduced sensitivity to the choice of pseudo-time step dt.
- Regularization: Line 47 implements the regularization described in Eq. (24).

Given that the hypersurface is mapped at the element nodes, the density at the cut structural boundary is computed using an approximate statistical approach, as implemented in lines 48–52.

For single-material compliance minimization of 3D structures, a 99-line MATLAB code is provided (see Appendix C). The code is executed in the same manner as the 2D single-material case using the following command:
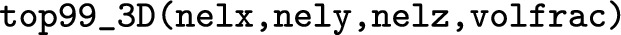


The update mechanism for the 3D structures is entirely consistent with that of the optimization code for single-material 2D structures; thus, further elaboration on this aspect is unnecessary herein.

## Numerical example

### Cantilever beam with composite material

**Fig. 5:**
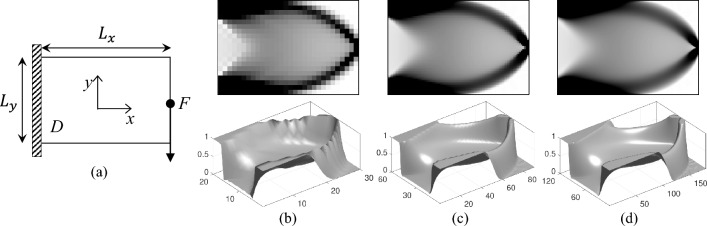
Comparison of optimized composite results for the 2D cantilever beam design with different discretizations. **a** Illustration for the cantilever beam problem. **b**–**d** present the optimized result with mesh of $30\times 20$, $90\times 60$, and $180\times 120$, respectively. The top figures are converged composite material distributions, and the bottom results are corresponding hypersurfaces

In this section, the 44-line MATLAB code for compliance minimization of 2D structures with composite materials is validated using a cantilever beam example. The configuration of the cantilever beam is depicted in Fig. 5a. The cantilever beam is fixed at its left end, with a concentrated load *F* applied at the midpoint of its right end. Three mesh discretization cases for the rectangular design domain *D* are investigated: $30\times 20$, $90\times 60$, and $180\times 120$ regular quadrilateral elements of the normalized dimensions of $L_{x}$ and $L_{y}$, with a material volume fraction constraint set at $50\%$ of the total design domain.

Figure 5 presents the optimized composite material distributions for the cantilever beam under three different mesh discretizations. The results demonstrate a consistent material distribution across the varying meshes. The computed compliance values for the optimized designs are 28.36, 29.09, and 29.55, corresponding to the $30\times 20$, $90\times 60$, and $180\times 120$ elements, respectively. As the mesh is refined, the hypersurface exhibits increased smoothness. This numerical example underscores the efficacy of the differential equation-based evolution method in achieving reliable and robust results within the framework of density-based topology optimization.

### MBB beam with single-material

**Fig. 6:**
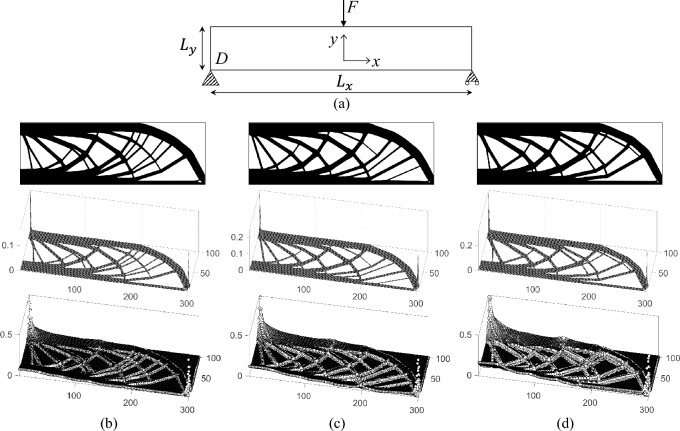
Comparison of optimized composite results for the 2D MBB beam design with different cases of penalty. **a** Illustration for the MBB beam **b**–**d** present the optimized result with penalty of 2, 3, and 4, respectively. The top figures are converged single-material distributions, the middle figures are the corresponding hypersurfaces, and the bottom results are corresponding sensitivity distributions

In the case of single-material compliance minimization, a 2D MBB beam example is investigated using the 55-line MATLAB code. As depicted in Fig. 6a, the beam is supported at the bottom left corner (fixed in both directions) and at the bottom right corner (restricted in the $y-$direction). A concentrated load *F* is applied at the midpoint of the top edge. The rectangular design domain *D* has normalized dimensions of $L_{x}=300$ and $L_{y}=100$, and the FEA mesh is discretized into $300\times 100$ regular quadrilateral elements. The material volume fraction is constrained to $50\%$. To investigate the impact of different penalty factors on the optimization results, three cases are considered: $p=2$, $p=3$, and $p=4$. The comparison of these cases provides insights into how the choice of the penalty factor influences the convergence and material distribution in the optimized design.

**Fig. 7:**
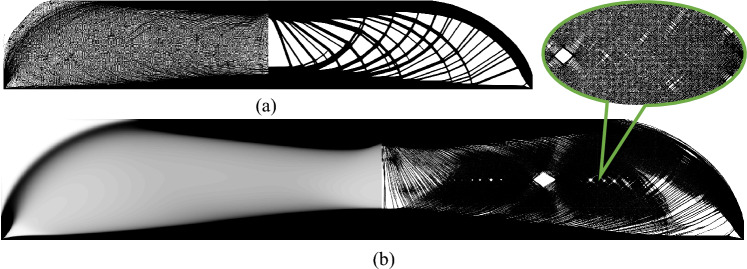
Boundary penalization effect. **a** Comparion of optimized results without penalization (left) and with penalization (right). The FEA mesh employed in the optimization is $600\times 200$, with 500 volume relaxation steps applied. **b** Comparion of optimized results of composite material (left) and single-material (right) with penalty $p=1+10^{-10}$. The FEA mesh employed in the optimization is $6000\times 2000$, with 2500 volume relaxation steps applied

The optimized results for the MBB beam problem are presented in Fig. 6. These results illustrate the effectiveness of the differential equation-based update scheme for density methods in the single-material case. For various penalty factors, the optimization results demonstrate consistency in material distribution (Fig. 6b–d). For the three penalty cases, the normalized compliances—computed with $p=1$ and recomputed FEA results for the finally converged structure—are 179.73, 180.45, and 180.16, respectively. The hypersurface remains predominantly flat except in the regions near the applied load and supports. This behavior contrasts with the composite material case, as the hypersurface for single-material design indicates only the presence or absence of material. As the penalty factor increases, the sensitivity differentiation becomes more pronounced. Penalization affects primarily boundary elements with intermediate densities, stabilizing the evolution of the structural boundaries. Figure 7a compares results with and without penalization. Without penalization, the optimization process fails to generate a distinct structural topology, whereas penalization ($p=2$) achieves a stable and well-defined material layout. Notably, even a marginal increase in the penalty factor (e.g., $10^{-10}$) significantly reduces oscillatory behavior in the optimization process. When the penalty factor approaches exact 1, the problem becomes nearly convex, enabling the solution to approximate the global optimum. Figure 7b contrasts the optimized composite material result (global optimum) with the single-material result for $p=1+10^{-10}$. The single-material result exhibits fiber-like microstructures, with normalized compliances of 179.87 and 188.23 for the composite and single-material cases, respectively, resulting in an offset of approximately $4.6\%$. Despite the close approximation to the global optimum, low penalization values may still induce oscillations and produce overly complex topologies that are impractical for manufacturing. Consequently, the penalty factor must be chosen judiciously. Based on these observations, the optimal range for the penalty factor is generally between 2 and 4.

**Fig. 8:**
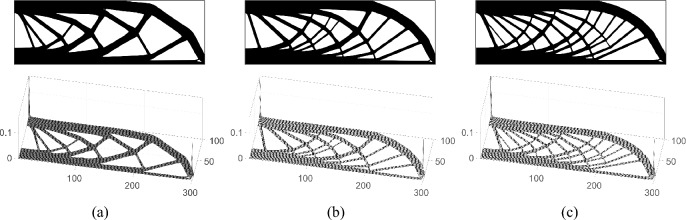
Comparison of optimized single-material results for the 2D MBB beam design with different material volume relaxation steps. **a**–**c** present the optimized result with volume relaxation step of 20, 40, and 80, respectively. The top figures are converged single-material material distributions, and the bottom results are corresponding hypersurfaces. After optimization convergence, the compliances are 180.88, 180.02, and 180.09, respectively

The influence of the material volume relaxation parameter $N_{r}$ on the optimized single-material results is analyzed in Fig. 8. The figure compares the optimized designs for the MBB beam problem under varying numbers of volume relaxation steps. As illustrated, a smaller number of relaxation steps results in simpler topological configurations. Conversely, as the number of relaxation steps increases, the converged layouts display enhanced structural complexity. This indicates that introducing an adequate number of relaxation steps helps slow down convergence, mitigates premature optimization, and facilitates the emergence of more refined structural details. However, upon comparing the objective function, it can be noted that although more complex structural details enhance structural stiffness, the potential for such enhancement is rather limited.

### 3D cantilever beam with single-material

**Fig. 9:**
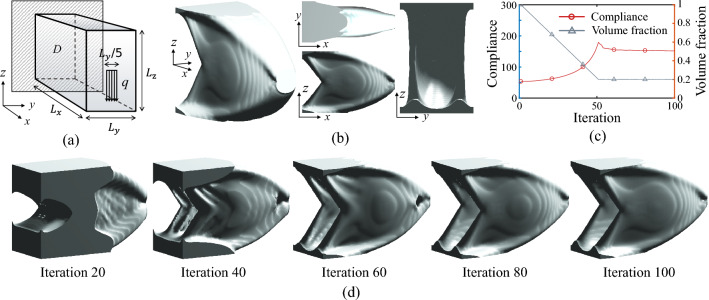
Optimized results of a single-material 3D cantilever beam design. **a** Schematic of the cantilever beam configuration. **b** Alternative view perspectives of the optimized structure. **c** Optimization iteration history. **d** Some intermediate results during the optimization process

For single-material compliance minimization, a 3D cantilever beam example is investigated using the 99-line MATLAB code. As depicted in Fig. 9a, the 3D beam is supported at the left end. A distributive load *q* is applied at the midline of the right end. The design domain *D* has normalized dimensions of $L_{x}=90$, $L_{y}=40$, and $L_{z}=60$, and the FEA mesh is discretized into $90\times 40\times 60$ regular hexahedral elements. The material volume fraction is constrained to $20\%$.

The 99-line MATLAB code for 3D single-material case is called using the following command:
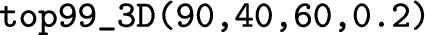

Figure 9b presents the optimized results. As observed from various perspectives, the optimized structure is a continuous and pore-free plate-shell structure. This type of structure effectively avoids stress concentration and enhances material utilization efficiency, thereby exhibiting high structural stiffness [29]. Figure 9c presents the iterative history curve of the optimization process. As observed, the optimization exhibits high convergence efficiency. Figure 9d shows the intermediate solutions at selected stages of the optimization process. It can be seen that during optimization, as the material volume decreases, voids form in the structure. However, as the optimization progresses, these voids gradually heal, ultimately resulting in a continuous plate-shell structure.

### Multiple load cases

**Fig. 10:**
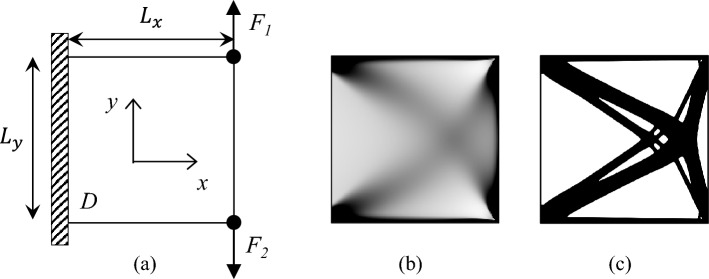
Multiple load cases problem. **a** Illustration of the problem definition. **b** Optimized result with composite material. **c** Optimized result with single-material

The proposed algorithm can be readily extended to address multiple loading scenarios. For instance, the two-load case problem illustrated in Fig. 10a can be solved with minimal modifications to the developed code. the developed codes can address it by changing several lines. The design domain *D* has normalized dimensions of $L_{x}=150$ and $L_{y}=150$, and the FEA mesh is discretized into $150\times 150$ regular quadrilateral elements. The material volume fraction is constrained to $40\%$.

For the composite material scenario, the following changes are made to the 44-line MATLAB code:

- Load vector definition: replace lines 18–19 with the following:

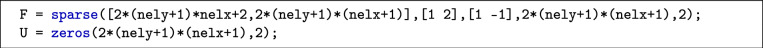

- FEA for multiple loads: modify line 26 to account for equilibrium equations for both load cases:

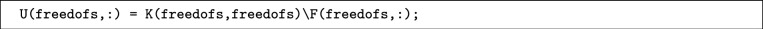

- Objective and sensitivity: replace lines 28–29 with the following:

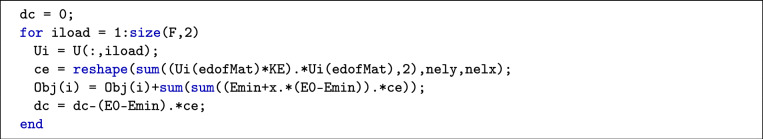


Using these adjustments, the optimized design in Fig. 10b can be achieved with the function call:
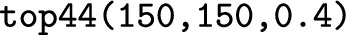


For the single-material scenario, the modifications to the 55-line code are analogous to those for the 44-line code, including changes to force boundary conditions, displacement vectors, FEA, and the objective function and sensitivity lines. The optimized design shown in Fig. 10c is obtained via:
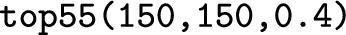


The optimized designs for composite and single-material cases yield compliances of 55.15 and 60.69, respectively. The single-material results exhibit consistency with prior work in [4]. However, due to the minimal filtering radius employed in Eq. (22), the optimized single-material design includes finer structural details and generally achieves a better objective function.

### Passive region

**Fig. 11:**
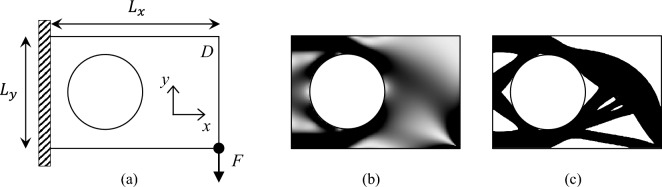
Multiple load cases problem. **a** Illustration of the problem definition. **b** Optimized result with composite material. **c** Optimized result with single-material

The proposed algorithm can be adapted to handle optimization problems involving passive regions with minor modifications. For instance, Fig. 11a illustrates a cantilever beam with a circular passive hole. The passive hole is treated as a fixed void (density value of zero) throughout the optimization process. The design domain *D* has normalized dimensions of $L_{x}=150$ and $L_{y}=100$, and the FEA mesh is discretized into $150\times 100$ regular quadrilateral elements. The material volume fraction is constrained to $50\%$. The passive circular region, with a radius of *nely*/3 and center at (*nely*/2 , *nelx*/3), is excluded from the optimization.

To address the passive region in the composite material scenario, the following changes need to be implemented:

- Insert the following lines after line 21:

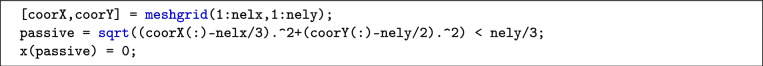

- The line below should be inserted after line 37:

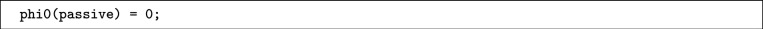

- For the single-material scenario, the following lines are added after line 22:

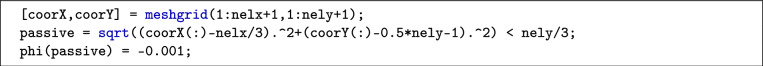

- The subsequent line is inserted after line 42:

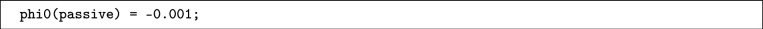


With these modifications, the 44-line and 55-line codes can generate the optimized designs shown in Fig. 11b and c, respectively. The results demonstrate the algorithm’s flexibility in handling passive regions.

### Different sampling mesh

By constructing an independent sampling grid and establishing an interpolation relationship between the sampling grid and the FEA mesh, the mesh dependency issue in design can be effectively mitigated.

For example, in the case of single-material, to accommodate the independent sampling mesh, the following changes are made to the 44-line MATLAB code:

- Replace line 22 with the following:

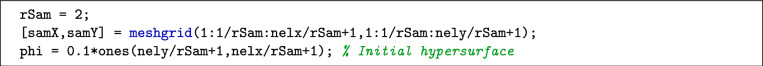


Herein, rSam denotes the ratio of the finite element mesh to the sampling mesh. For example, when the FEA mesh is $300\times 100$ and the sampling mesh is $150\times 50$, then, rSam=2.

- Insert the the following line after line 33:

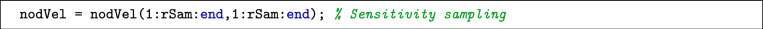

- Replace line 43 with the following:

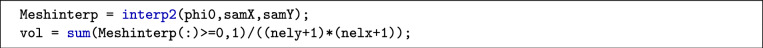

- Replace line 48–51 with the following:

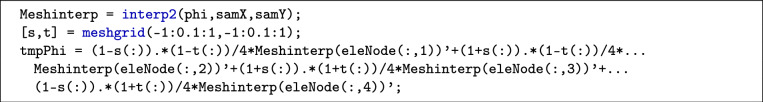


After these modifications, independent sampling mesh can be achieved, and the relationship between the sampling mesh and FEA mesh can be established using interpolation.

**Fig. 12:**
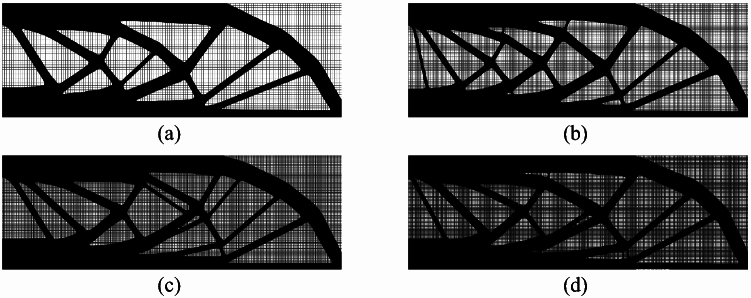
Optimized structures of a MBB beam under identical sampling grids but varying FEA meshes. **a** Sampling grid: $150\times 50$; FEA mesh: $150\times 50$. **b** Sampling grid: $150\times 50$; FEA mesh: $300\times 100$. **c** Sampling grid: $150\times 50$; FEA mesh: $450\times 150$. **d** Sampling grid: $150\times 50$; FEA mesh: $600\times 200$

For instance, regarding the MBB beam problem shown in Figs. 6a, 12 compares the optimized results of this MBB beam under three distinct mesh configurations. With a fixed sampling grid of $150\times 50$, four analysis meshes—$150\times 50$, $300\times 100$, $450\times 150$, and $600\times 200$—are considered under identical conditions. It can be observed that, despite the progressive refinement of the analysis mesh, the optimized structure does not exhibit increased structural details with mesh densification due to the unchanged sampling grid, thereby demonstrating a certain degree of mesh independence.

### Sensitivity comparison

In the proposed method, the sensitivity driving the evolution of the hypersurface can be constructed based on diverse gradient information, such as element derivatives or topological derivatives. When constructing the sampled gradient information using topological derivatives, this can be achieved by implementing the following modifications to the 55-line MATLAB code:

- Add the following lines after line 14:

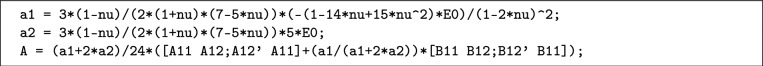

- Replace line 26 with the following:

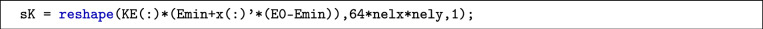

- Replace line 30–33 with the following:

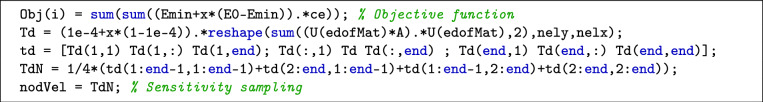


**Fig. 13:**
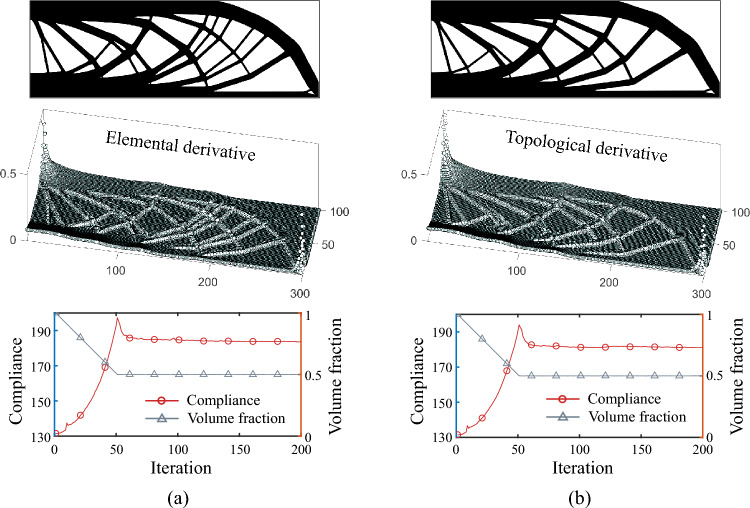
Optimized results of a MBB beam under different gradient information. **a** Optimized results using element derivatives. Top: optimized structure; Middle: elemental derivative distribution; Bottom: optimization iteration history. Normalized compliance: 179.73. **b** Optimized results using using topological derivatives. Top: optimized structure; Middle: elemental derivative distribution; Bottom: optimization iteration history. Normalized compliance: 181.13

With these modifications to the 55-line codes, optimized designs using topological derivative can be generated. For example, for the MBB example shown in Fig. 6a, the comparison for optimized results using elemental and topological derivatives is displayed in Fig. 13. It can be observed that the optimized results obtained using topological derivatives and element derivatives exhibit consistency, with both the distribution of derivatives and the iterative convergence process being relatively consistent.

### Update scheme comparison

The relative and absolute increment schemes of the OC method exhibit different characteristics. Here, a cantilever beam benchmark case is employed to conduct a comparison between these two schemes. The boundary conditions for the cantilever beam is depicted in Fig. 5a. The rectangular design domain *D* is discretized with $150\times 100$ regular quadrilateral elements of the normalized dimensions of $L_{x}$ and $L_{y}$, with a material volume fraction constraint set at $50\%$ of the total design domain. For the relative increment scheme of the OC method, the standard code of top88 in [4] was employed with penal=2, rmin = 1.5, and ft = 1 (sensitivity filtering). Also, the relative increment scheme uses a damping function with the damping factor $\beta =1/2$ while updating the design [4]. For the absolute increment scheme of the OC method, the code of top55 was employed with penal=2.

**Fig. 14:**
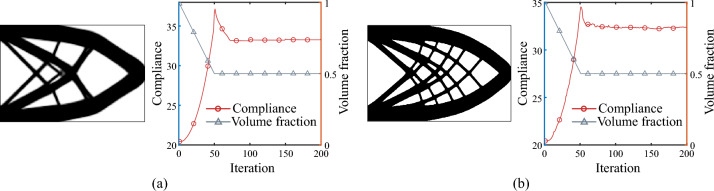
Comparison of optimized results under different OC update schemes. **a** Results using the OC relative increment scheme. Top: optimized structure; Bottom: optimization iteration history. Normalized compliance: 31.78. **b** Results using the OC absolute increment scheme. Top: optimized structure; Bottom: optimization iteration history. Normalized compliance: 31.42

Figure 14 compares the optimized results of the two OC update schemes. As observed, for the relative increment scheme, the adoption of sensitivity filtering and update damping leads to more stable and efficient convergence during optimization; however, the optimized configuration tends to be structurally simpler. In contrast, the absolute increment scheme exhibits minor oscillations throughout the convergence process, yet its optimized results feature richer geometric details and superior performance. Therefore, the relative increment scheme of the OC method is characterized by high efficiency and stability, though it sacrifices some precision in gradient information response. Conversely, the absolute increment scheme demonstrates heightened sensitivity to gradient variations, enhancing its ability to identify optimal solutions, albeit with potential oscillatory behavior during the optimization process.

**Fig. 15:**
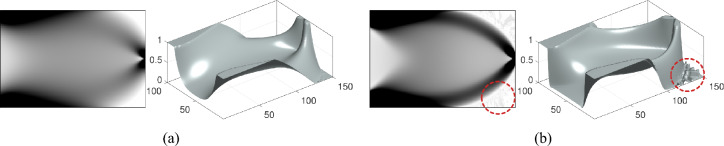
Influence of time step size on the optimized results of a composite cantilever beam design. **a** For smaller time step sizes, the optimization process converges slowly with low efficiency. **b** For larger step sizes, although the optimization converges more rapidly, numerical instability characterized by optimization oscillations emerges

When performing design updates using the OC absolute increment scheme, careful consideration must be given to the step size parameter. When the step size is excessively small, design updates proceed slowly, leading to low optimization efficiency; when the step size is too large, the optimization process is prone to premature convergence, accompanied by oscillations. As illustrated in Fig. 15, for the cantilever beam optimized using composite materials, when an excessively small step size is selected, structural updates occur at a slow pace. When a relatively large step size is chosen, oscillations persist in the structural boundary regions even after approaching convergence. These oscillations, on the one hand, reflect the high sensitivity of the OC absolute increment scheme to gradient information, which aids in escaping local optimal solutions. However, excessively intense oscillations may also lead to optimization failure. Therefore, selecting an appropriate step size is crucial when using the OC absolute increment scheme for design updates.

## Discussion

### Numerical instability

Although the OC absolute increment scheme is more sensitive to gradient information and enables rapid capture of gradient variations, facilitating the identification of superior solutions, it is prone to oscillations during the optimization process. Numerical instabilities may emerge when using differential equations, particularly due to the absolute increment format inherent in the method. Ensuring stability requires careful consideration of both the hypersurface and velocity aspects. If the hypersurface function is smooth, and the velocity governing the evolution of this surface is similarly smooth, the resulting hypersurface representing the material distribution after optimization will converge in a smooth manner. Hence, preventing oscillations in the optimization process can be approached by managing these two factors effectively.

### Hypersurface regularization

To maintain a desirable morphology of the hypersurface, regularization techniques are often necessary. These methods may involve controlling the evolution of the hypersurface through move limits, managing its curvature, and scaling or filtering the magnitudes of the hypersurface. Regularization ensures that the hypersurface evolves in a way that accommodates changes in the sensitivity field, ultimately promoting efficient and stable convergence of the optimization process.

### Sensitivity regularization

Since the sensitivity field serves as the primary driver for the evolution of the hypersurface, it is often subject to regularization. In some cases, sensitivity can exhibit significant fluctuations even with small changes in material properties, potentially leading to numerical instability. To maintain the well-posedness of the sensitivity field, regularization techniques, such as filtering, can be employed to ensure smoothness. Filtering strategies for sensitivity can include the following approaches:

- Spatial domain filtering: This method regularizes the sensitivity field by averaging the sensitivity at a given point with those of its neighboring points within the same spatial domain. A typical example in density-based methods is the convolution scheme, which functions as a form of transverse filtering.
- Time domain filtering: In contrast to spatial filtering, time domain filtering adjusts the sensitivity at a point based on its own historical values, acting as a form of vertical filtering. This approach is particularly useful in preventing abrupt changes or mutations in the sensitivity field.
- Frequency domain filtering: When the design problem is transformed into the frequency domain, filtering techniques can be applied to modulate the sensitivity field by adjusting specific frequency components.

Given the flexibility of filtering methods depending on the problem at hand, various filtering strategies or combinations can be effectively combined to ensure the smoothness of the sensitivity field.

## Conclusion

In this paper, a differential equation-based evolution strategy is proposed for density methods, with the differential equation derived from the generalized evolution of a hypersurface. By leveraging the orthogonal component of the generalized governing equation, an absolute increment formulation of the OC method is developed. In contrast to the relative increment formulation, the absolute increment formulation is more active and responsive during the optimization process, which helps facilitate convergence toward improved solutions. Based on the differential equation-based update scheme, a 44-line MATLAB code for composite material, a 55-line MATLAB code for 2D single-material optimization, and a 99-line MATLAB code for 3D single-material optimization are developed, specifically addressing the compliance minimization problem. Numerical examples demonstrate that the proposed update scheme effectively solves density distribution optimization problems, and the algorithm can be easily adapted to various other problems.

However, a key limitation of the evolution strategy based on differential equations lies in the potential numerical instability that may arise during the optimization process. To ensure stable convergence, careful attention must be paid to the selection of the time step size, and regularization techniques are applied to both the hypersurface and sensitivity fields. The core idea behind these regularizations is to maintain the smoothness of both the hypersurface and sensitivity, thereby enhancing the stability and performance of the optimization process.

## Data Availability

No datasets were generated or analysed during the current study.

## Declarations

### Replication of results

To replicate the results presented in this work, readers can refer to the MATLAB code provided in the Appendix. Additionally, the source MATLAB code is available for download on GitHub.: https://github.com/MCM-QMUL/StruOptComp
